# Effect of artificial insemination, ruminal incubation, and esophageal tubing on cortisol concentration in blood of lactating dairy cows

**DOI:** 10.3168/jdsc.2024-0676

**Published:** 2024-11-05

**Authors:** Victoria Ferreira, Gonzalo Ferreira

**Affiliations:** 1 Marymount School of New York, New York, NY 10029; 2 School of Animal Sciences, Virginia Tech, Blacksburg, VA 24061

## Abstract

Cortisol is a hormone associated with pain, fear, distress, or discomfort. We hypothesized that human interventions increase cortisol concentrations in dairy cow plasma. Therefore, the objective of this study was to determine the concentration of cortisol in plasma before and after animals were subjected to routine on-farm and research procedures, including artificial insemination, ruminal incubation, and esophageal tubing. This study used 40 lactating Holstein cows. Experimental treatments consisted of 4 interventions. Before any intervention, a first blood sample was collected from all cows. Following this blood sample, all cows were assigned to 1 of 4 treatments. A negative control treatment consisted of collecting a second blood sample at least 30 min after the previous one, without any human intervention. Artificial insemination was performed by the herd managers from the Virginia Tech Dairy Complex (Blacksburg, VA). A rumen incubation treatment consisted of collecting a second blood sample 30 min after opening the rumen cannula, inserting the operator's arm, and pulling ruminal contents out of the rumen for 2 min, mimicking a ruminal in situ incubation, and replacing the cannula plug. The esophageal tubing treatment consisted of collecting a second blood sample 30 min after inserting an esophageal tube into the esophagus for 2 min, mimicking a ruminal drenching procedure. Blood samples were collected from the coccygeal vessels. Cortisol concentration in plasma was measured by an independent laboratory using a chemiluminescence assay. The experiment was designed as a completely randomized design with repeated measures, where cow was the subject and the pre- and postintervention sampling were the repeated observations. The statistical model included the fixed effect of treatment, the random effect of cow, the fixed effect of time, the fixed effect of the treatment by time interaction, and the random residual error. According to the Akaike information criterion, compound symmetry was used as the covariance structure for the repeated measures. Cortisol concentrations in plasma after the interventions did not differ from the concentrations in plasma before the interventions. Cows subjected to artificial insemination had greater cortisol concentrations than cows subjected to the other treatments. No interaction existed between treatment and time. Most cows subjected to insemination in a palpation rail had elevated cortisol concentrations before the intervention. Therefore, animal restraint seems to affect cortisol concentrations in plasma more than the actual human intervention. Overall, human interventions such as artificial insemination, ruminal incubation, and esophageal tubing did not elevate the cortisol concentrations in the plasma of lactating dairy cattle under the conditions of this study.

## Highlights

• Human interventions did not elevate the cortisol concentrations in plasma.
• Cows inseminated outside pens had higher cortisol concentrations before insemination.
• Animal restraint might affect cortisol concentrations more than human intervention.

## Graphical Abstract

**Figure:**
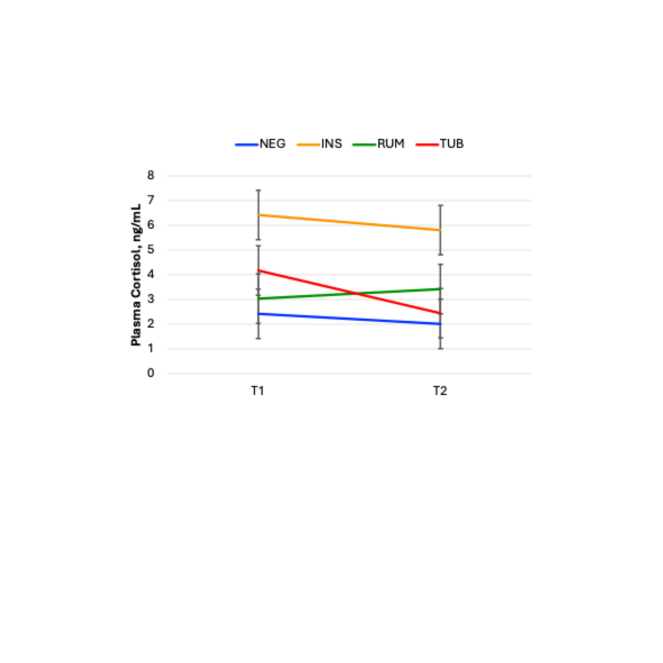
**Summary:** We determined the concentration of cortisol in plasma before (T1) and after (T2) animals were subjected to no intervention (NEG), artificial insemination (INS), ruminal incubation (RUM), or esophageal tubing (TUB). Blood samples from 40 lactating Holstein cows were collected from the coccygeal vessels to measure cortisol concentration. Cortisol concentrations in plasma after the interventions did not differ from those before the interventions. Cows subjected to artificial insemination had greater cortisol concentrations than cows subjected to the other treatments. No interaction existed between treatment and time. Some cows subjected to insemination in a palpation rail had elevated cortisol concentrations before the intervention. Therefore, animal restraint seems to affect cortisol concentrations in plasma more than actual human intervention. In conclusion, human interventions such as INS, RUM, and TUB did not elevate the cortisol concentrations in the plasma of lactating dairy cattle under the conditions of this study.

The social license to operate is the trust and acceptance of the production practices from stakeholders or communities (Pejman et al., 2019; Hampton et al., 2020; Rice et al., 2020). Because societal concerns exist about intensive animal production practices (Pejman et al., 2019) and the use of animals for research, the dairy industry must carefully enforce animal welfare in both production and research practices. Animal welfare standards imply that we guarantee freedom from pain and injury, freedom from hunger and thirst, freedom from discomfort, freedom from fear and distress, and freedom to express normal behavior (Pejman et al., 2019). Assessing pain, fear, distress, or discomfort is challenging without adequate quantification tools. The lack of tools to quantify pain, fear, distress, or discomfort may lead consumers to subjective interpretations of pain, fear, distress, or discomfort.

Cortisol, also known as the stress hormone, is a hormone associated with pain, fear, distress, or discomfort. The hypothalamic-pituitary-adrenocortical axis, which regulates the release of cortisol, may be activated in response to these stressors, therefore elevating the concentration of cortisol in the blood (Cook et al., 2000; Dickerson and Kemeny, 2004). For example, McMeekan et al. (1998) observed that cortisol concentration in plasma increased from 19 to >50 ng/mL within 20 min when 3-mo-old dairy female calves were dehorned without anesthesia. Similarly, Martin et al. (2022) reported that cortisol concentration in serum increased from ∼9 to ∼25 ng/mL within 45 min when 4-mo-old dairy male calves were castrated without anesthesia. Measurement of cortisol concentrations in blood could be a useful tool to objectively assess the absence or presence of pain, fear, distress, or discomfort when dairy cows are subjected to certain human interventions (Koenneker et al., 2023), such as artificial insemination, ruminal incubation, or esophageal tubing. In this study, we hypothesized that human interventions would increase cortisol concentrations in plasma relative to baseline concentrations measured before the intervention. Therefore, the objective of this study was to determine the cortisol concentration in plasma before and after animals were subjected to artificial insemination, ruminal incubation, or esophageal tubing.

All procedures involving cow handling were performed following the ethical guidelines and with the approval of the Virginia Tech Institutional Animal Care and Use Committee (IACUC #22-146). We used 40 lactating Holstein cows (2.5 ± 1.5 lactations and 225 ± 76 DIM) from the dairy herd of the Virginia Tech Dairy Complex (Blacksburg, VA), which occurred between June 24 and July 12, 2024. Ten replicates per treatment was the most convenient sample size for executing the experiment within the allocated timeframe and budget. The lactating cows in this study were housed in pens within a freestall barn with 2 rows of stalls bedded with sand, headlocks at the feedbunk, and 2 water troughs per pen. The stocking density (i.e., cows per stall) was 100%. Heat stress was managed with automatic fans (1.2-m diameter) positioned over the stalls and feeding alleys. Cows were milked twice daily (0100 h and 1200 h) in a double-12 parallel milking parlor. Cows were fed a TMR composed of (DM basis) 42% corn silage, 8% grass hay, and 50% concentrate once daily (0800 h), and feeding was ad libitum (<5% refusals). The manure from the stalls was scraped at every milking by personnel while herding cows to the milking parlor. The manure from the walking alleys within the pen was removed periodically during the day (>4 times per day) using an automatic flushing system with recycled water. New or recycled sand was added every week.

Experimental treatments consisted of 4 human interventions: a negative control (**NEG**) in which the animal was not handled, artificial insemination (**INS**), ruminal incubation (**RUM**), or esophageal tubing (**TUB**). Before any intervention, a first blood sample (**T1**) was collected from all cows to act as a baseline. Following the baseline blood sampling, cows were subjected to 1 of the 4 treatments. Cows allocated to the NEG and TUB treatments were randomly selected as they voluntarily locked themselves in the headlocks during feeding or after milking. To ensure adequate basal cortisol concentrations, blood samples were collected from a group of 10 to 15 consecutive cows, and sampling time was recorded. Then, every other cow was assigned to the TUB treatment. Cows with existing ruminal cannulas were arbitrarily allocated to the RUM treatment. These RUM cows voluntarily locked themselves in the headlocks during feeding or after milking. Lactating cows with “do not breed” (**DNB**) status (n = 6) and lactating cows scheduled to be inseminated on June 28, 2024 (n = 4) were arbitrarily allocated to the INS treatment. The DNB cows within the INS group voluntarily locked themselves in the headlocks during feeding or after milking. The cows scheduled for insemination within the INS group were sorted and driven to a palpation rail outside the pen by 2 herd managers. Cow sorting and placement in the palpation rail concluded by 0645 h. The NEG treatment consisted of collecting a second blood sample (**T2**) after waiting at least 30 min following the baseline sampling, without any human intervention. The INS treatment consisted collecting a second blood sample 30 min after inseminating the cow. Artificial insemination was performed by 2 herd managers from the Virginia Tech Dairy Complex. The RUM treatment consisted of collecting a second blood sample 30 min after opening the rumen cannula, inserting the operator's arm, pulling ruminal contents out of the rumen for 2 min, mimicking a ruminal in situ incubation (Ferreira et al., 2022), and replacing the cannula plug. The TUB treatment consisted of collecting a second blood sample 30 min after inserting an esophageal tube (500 Cattle Pump System, Springer Magrath, Glencoe, MN) into the esophagus for 2 min, mimicking a ruminal drenching procedure. Both RUM and TUB procedures were performed by the principal investigator (GF). Artificial insemination is a common practice in the dairy industry that is sometimes subjected to consumers' scrutiny. Ruminal incubation is a common practice in ruminant nutrition studies. The use of esophageal tubing is a common practice in treating sick animals. Because it generates distress to the animal (i.e., the animal rejects or “fights back” against the intervention), we presumed and hypothesized that TUB served as a positive control.

Blood samples were collected by the principal investigator (GF) in 4 sets while cows were restrained with activated restraining headlocks. Sampling occurred in 4 sets of 10 cows each to avoid cows being locked in the headlocks for and extensive amount of time. Two sets of cows were sampled in the afternoon after returning to the pen from the milking parlor. Another 2 sets of cows were sampled in the morning immediately after feed delivery. All cows in the INS treatment were sampled during 2 mornings. Cows in the NEG, RUM, and TUB treatments were randomly sampled in the morning or afternoon. In most cases, cows voluntarily accessed the headlocks; however, 4 cows were handled in a palpation rail for artificial insemination, and 1 cow was handled while standing in the stall, also for insemination. Blood samples were collected from the coccygeal vessels using 20-gauge needles attached to a Vacutainer (Becton, Dickinson and Company) containing sodium heparin. The blood sampling procedure consisted of raising the tail of the cow, scrubbing the tail area with a cotton round soaked in ethanol, inserting the needle into the tail in a caudo-dorsal direction, attaching the Vacutainer to the needle, waiting until at least half of the Vacutainer was full of blood, and then scrubbing and pressing the tail area with another cotton round soaked in ethanol. Collected blood samples were immediately placed in ice, and samples remained in ice for less than 90 min. After a 10-min centrifugation at 2,000 × *g* and room temperature, plasma was extracted, transferred to a microcentrifuge tube, immediately frozen (−20°C), and submitted to the Animal Health Diagnostic Center at Cornell University (Ithaca, NY) for cortisol analysis by chemiluminescent assay on a Immulite 2000 XPi system for cattle (Siemens Healthcare Diagnostics Inc., Tarrytown, NY). The informed intra- and interassay coefficients of variation were 8.0% and 7.6% respectively.

The experiment was designed as a completely randomized design with repeated measures, where cow was the subject and T1 and T2 were the repeated observations. The statistical analysis was performed using the MIXED Procedure of SAS (SAS version 9.4, SAS Institute Inc., Cary, NC). To test human interventions and their effect, the model included the fixed effect of treatment (3 df), the random effect of cow (36 df), the fixed effect of time (1 df), the fixed effect of the treatment by time interaction (3 df), and the random residual error. To test the 2 insemination alternatives within the INS treatment and their response, the model included the fixed effect of treatment (1 df), the random effect of cow (9 df), the fixed effect of time (1 df), the fixed effect of the treatment by time interaction (1 df), and the random residual error. According to the Akaike information criterion, compound symmetry was used as the covariance structure for the repeated measures. When treatments were significantly different, comparisons among treatments were performed using Tukey's method.

In this study, we hypothesized that the cortisol concentration in plasma would increase after subjecting animals to human interventions such as INS, RUM, or TUB. Contrary to our hypothesis, the mean cortisol concentration in plasma after the interventions did not differ from the cortisol concentration in plasma before the interventions (*P* = 0.22) in any of the treatments (Figure 1). Cows subjected to INS, however, had more elevated concentrations of cortisol in plasma (6.1 ng/mL; *P* < 0.03) than cows subjected to the other treatments (2.2, 3.2, and 3.3 ng/mL for NEG, RUM, and TUB, respectively). No interaction existed between treatment and time (*P* = 0.47).

**Figure 1:**
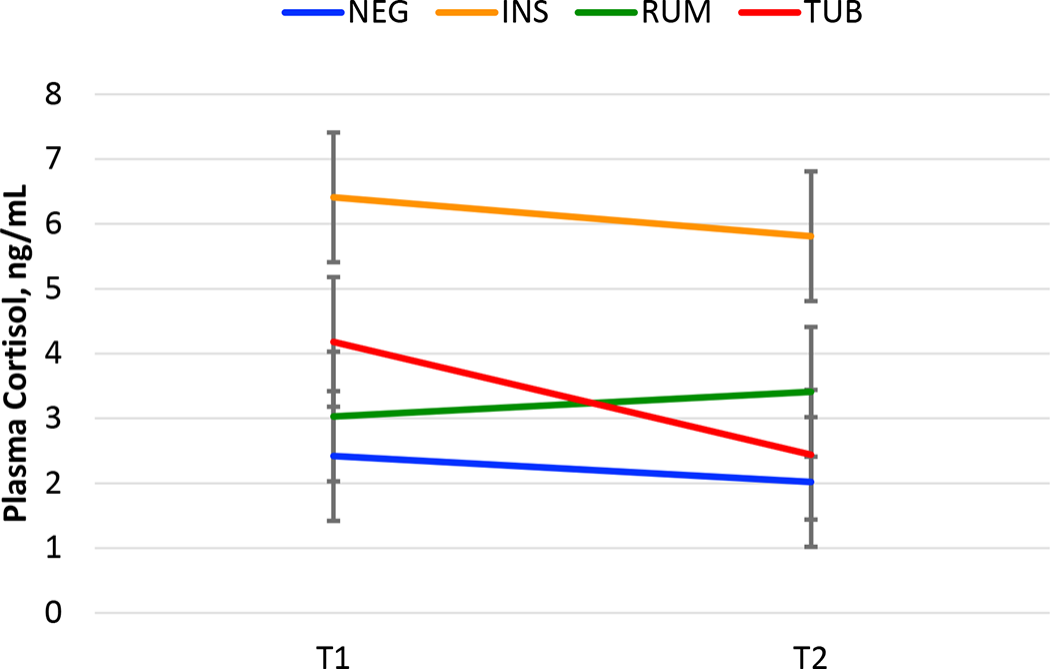
Mean cortisol concentration (ng/mL) in plasma from lactating dairy cows subjected to different human interventions (NEG: negative control with no intervention, n = 10; INS: artificial insemination, n = 10; RUM: ruminal incubation, n = 10; and TUB: esophageal tubing, n = 10). We obtained a baseline concentration (T1) before the intervention and a postintervention concentration (T2) 30 min after the intervention. Human intervention (*P* = 0.03), time (*P* = 0.22), interaction (*P* = 0.47). Bars indicate SEM.

After subjecting cows to human interventions, we expected cortisol concentrations to increase relative to baseline levels. Koenneker et al. (2023) reported elevated cortisol concentrations in plasma 40 min after finishing hoof trimming. Based on that study (Koenneker et al., 2023), we drew blood twice with a 30-min interval between the human intervention and the second sampling. It is plausible, however, that cortisol concentrations returned to baseline levels in less than 30 min. Therefore, in future studies, blood samples should be collected more frequently and, perhaps, also during the human intervention (Koenneker et al., 2023).

According to Fischer-Tenhagen et al. (2018, p. 5439), “handling and restraining of dairy cattle, however, has been shown to rapidly increase concentration of cortisol in plasma. . . .” In our study, 35 of the 40 cows voluntarily accessed the headlocks while eating during feeding time or after returning from the milking parlor, and we consider minimal restraining stress, at least for the cows subjected to the NEG, RUM, and TUB treatments. Differently, cows subjected to INS had elevated cortisol concentrations in blood at T1 and T2 (Figure 1) in our study. Our data suggest, therefore, that human interventions did not trigger an elevation of cortisol concentration or, alternatively, that the return to basal levels occurred within 30 min of the intervention.

The elevated cortisol concentration for INS cows is intriguing, and it appears that a cortisol surge was triggered by T1. Fischer-Tenhagen et al. (2018) based their statement on work from Cook et al. (2000). Cook et al. (2000), however, based their statement on one study with wild white-tailed deer (Seal et al., 1972) and another study with wild buffaloes and Nguni cattle in South Africa (Hattingh et al., 1988). Seal et al. (1972) guided deer onto a runway leading into a box trap and placed blindfolds and leg shackles before drawing blood. This process took 5 to 10 min (Seal et al., 1972). Hattingh et al. (1988) reported high cortisol concentrations in plasma from Nguni cattle restrained in a chute but also reported lower cortisol concentrations in plasma from unrestrained Nguni cattle. Studies such as Seal et al. (1972) and Hattingh et al. (1988), as well as the review from Grandin and Shivley (2015), provide a valuable perspective on restraining stress and help to understand the elevated cortisol concentration in cows subjected to INS. As described before, we inseminated 4 of the 10 cows following a timed artificial insemination protocol while we restrained the cows in a palpation rail outside the freestall pen. Figure 2 shows elevated cortisol concentrations at T1 in these 4 cows while being restrained in the palpation rail. Contrarily, the other 6 cows subjected to INS (Figure 2) had cortisol concentrations at T1 similar to cows subjected to the NEG treatment (Figure 1). The latter 6 cows were handled within the freestall barn while eating (n = 5) or standing in the sand-bedded stall (n = 1). Therefore, we attribute the elevated cortisol concentration at T1 to the restraining conditions or the status in the estrus cycle but not to the insemination as a human intervention. It is worth highlighting that the 6 cows bred in the freestall barn were classified as “do not breed” cows, meaning they were not eligible to become pregnant based on management decisions. At the moment of this study, we did not inspect for signs of estrus among these 6 cows. Therefore, we still do not know whether the elevated cortisol concentration from cows restrained in the palpation rail is explained by the animal restraint or by being in estrus. The confounding of these 2 factors warrants additional research.

**Figure 2:**
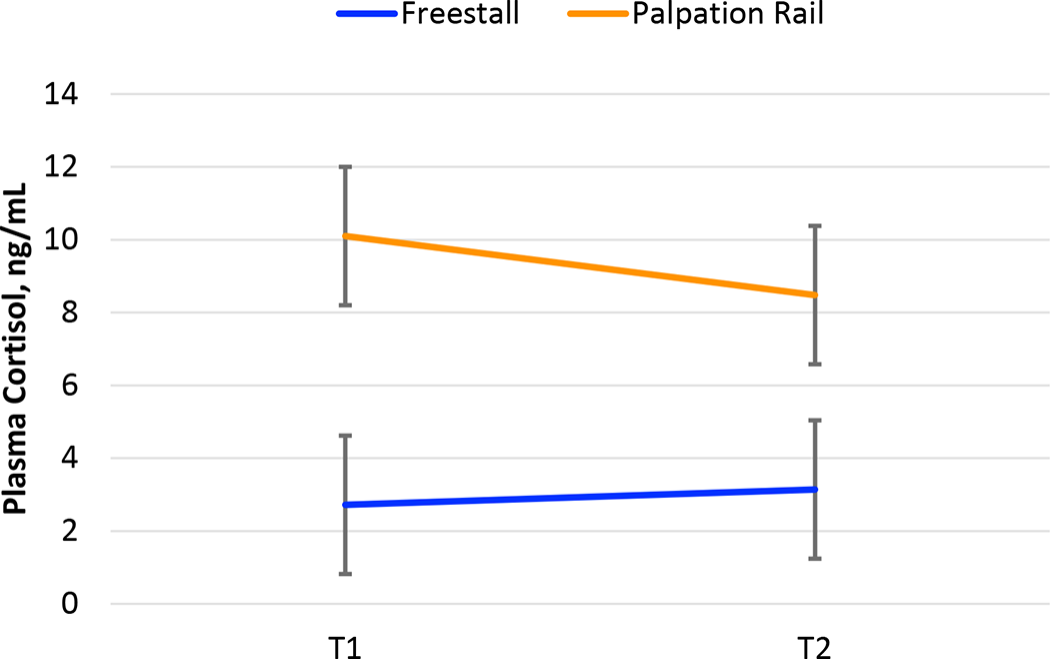
Mean cortisol concentration (ng/mL) in plasma from lactating dairy cows subjected to artificial insemination within a freestall barn (n = 6) or at a palpation rail (n = 4). We obtained a baseline concentration (T1) before the intervention and a postintervention concentration (T2) 30 min after the intervention. Cows in the palpation rail were subjected to insemination following a timed artificial insemination protocol. Cows in the freestall barn were not eligible to become pregnant (i.e., “do not breed” status) and not necessarily in estrus. Location of insemination (*P* = 0.04), time (*P* = 0.59), interaction (*P* = 0.36). Bars indicate SEM.

In conclusion, human interventions such as artificial insemination, ruminal incubation, and esophageal tubing did not elevate the cortisol concentrations in the plasma of lactating dairy cattle under the conditions of this study. We acknowledge, however, that we should replicate this experiment under a more frequent blood collection schedule to validate the impact of human interventions on cortisol concentration in plasma and animal welfare.
